# Post-kala-azar dermal leishmaniasis: insights into underlying pathogenic mechanisms and genetic landscape

**DOI:** 10.3389/fmicb.2026.1765586

**Published:** 2026-03-02

**Authors:** Soumyadeep Mukherjee, Shreya Karmakar, Vishal Kumar Singh, Rajiv Kumar, Shyam Sundar

**Affiliations:** 1 Department of Medicine, Institute of Medical Sciences, Banaras Hindu University, Varanasi, India; 2 Centre of Experimental Medicine and Surgery, Institute of Medical Sciences, Banaras Hindu University, Varanasi, India

**Keywords:** biomarkers, genomic studies, *Leishmania donovani*, post-kala-azar dermal leishmaniasis, visceral leishmaniasis

## Abstract

Post-apparently successful treatment visceral leishmaniasis (VL), caused by protozoan parasite *Leishmania donovani*, is often followed by a dermal manifestation among patients known as post-kala-azar dermal leishmaniasis (PKDL). Although non-fatal disorder PKDL manifests itself clinically with a spectrum of cutaneous lesions, including macular, papular, nodular, or polymorphic types, that appear following apparent cure from VL. The absence of reliable non-invasive diagnostic techniques contributes to the underreporting of PKDL, particularly in rural regions. Individuals affected by PKDL may act as reservoirs of *Leishmania*, posing a significant challenge to ongoing VL elimination initiatives. The transition from VL to PKDL is governed by a complex interplay between host immune mechanisms and parasite-specific genetic polymorphisms. Investigations into the molecular dialog between host and parasite employing both *in-vitro* and *in-silico* methodologies are currently underway to elucidate the underlying biological processes. A key objective of these efforts is the identification of reliable biomarkers associated with PKDL, which would facilitate a comprehensive understanding of disease progression and enable the development of improved diagnostic tools for early detection. In this context, genome sequencing has emerged as a critical tool for uncovering genetic variants of *L. donovani* that contribute to parasite persistence in a subset of individuals, even after effective VL therapy. Insights gained from genomic studies may also reveal novel therapeutic targets and inform vaccine development strategies, thereby opening new avenues for disease control and eradication. This review aims to examine the molecular strategies being employed to investigate the pathophysiology of PKDL, with an emphasis on portraying the mechanistic differences between VL and PKDL. A nuanced understanding of these distinctions is essential for effective disease management, early diagnostic intervention, and interruption of transmission cycles in endemic regions.

## 1 Introduction

The World Health Organization (WHO) classifies 20 different infectious diseases as neglected tropical diseases (NTDs). Despite their widespread distribution and potentially fatal outcomes, these diseases face negligence in terms of funds and inadequate attention from academia and pharmaceutical firms. Among NTDs, leishmaniasis is the second most prevalent after malaria (Chanda, 2021; Sundar et al., 2024). More than 1 million new cases are reported annually with majority of the affected population belonging to socioeconomically disadvantaged populations (EBioMedicine, 2023; WHO, 2025). Female sandflies of the genera of *Phlebotomus* (Old World) and *Lutzomyia* (New World) act as vectors responsible for spreading the disease. Approximately 20 different species of the protozoan parasite belonging to the subgenera *Leishmania* and *Viannia* of genus *Leishmania* are responsible for the wide range of clinical manifestations (Tiwari et al., 2024).

Clinical manifestations of leishmaniasis are broadly classified into three major forms, cutaneous leishmaniasis (CL), mucocutaneous leishmaniasis (MCL) and visceral form, depending on the species of *Leishmania* causing infection and complex interplay between host–parasite interactions. CL manifests as one or more skin lesions, typically on exposed regions such as the face, arms, and legs. These lesions usually start off as little bumps and then grow into nodules or ulcers over time. As per WHO 2025 report, there are more than 1 million new cases of CL per year (WHO, 2025; Kim et al., 2023). Even though the sickness normally goes away on its own, it can have long-lasting impacts on both mental and physical health, which includes scarring. The primary species that cause CL in the New World are *L. braziliensis*, *L. mexicana*, *L. amazonensis*, *L. panamensis*, *L. guyanensis*, and *L. peruviana* (Blaizot et al., 2024). The most frequent species in the Old World are *L. infantum*, *L. major*, *L. tropica*, and *L. aethiopica* (Pinheiro and De Souza, 2022). MCL, often known as espundia, is another kind commonly which produces awful lesions on the mucous membranes of the mouth, throat, and nose, which can lead to serious facial deformities (Roca and Roca, 2020). In the New World, MCL is far more widespread, notably in the South American belt that goes from Peru to Bolivia, Brazil, and Paraguay. Every year, there are roughly 1,700 new cases of MCL are registered. In Old World it is usually MCL arises from *L. major*, *L. infantum*, and *L. tropica* (Sundar et al., 2024). Of the three, visceral leishmaniasis (VL) also known as kala azar is the most severe form and can be fatal, if left untreated. Each year about 50,000 to 90,000 new cases of VL are reported (WHO, 2025; Kadayat et al., 2024). As of November 2025, a total of 52 VL-endemic countries had reported their 2024 data to the WHO Global Leishmaniasis Program. Notably, seven countries like Brazil, Ethiopia, India, Kenya, Somalia, South Sudan, and Sudan collectively contributed more than 85% of the global VL case burden for 2024 (WHO, 2025; Alvar et al., 2012). A non-fatal complication of VL is known as post kala-azar dermal leishmaniasis (PKDL), commonly develops in patients from South Asian and East African regions. Approximately 5–15% of patients, cured of VL in the Indian Subcontinent (ISC) whereas nearly 50% of those in East African region develops PKDL after treatment (Rahman et al., 2010; Ramesh et al., 2015; Zijlstra et al., 2003). The first description of PKDL in ISC was reported by UN Brahmachari in the year 1922 at Calcutta School of Tropical Medicine where he observed *Leishmania donovani* (LD) bodies in the dermal lesion, referring the condition as “dermal leishmanoid’ (Brahmachari, 1922). Clinically, PKDL manifests as skin eruptions in three forms, these are macules, papules, and nodules or as combination of three, generally referred as polymorphic lesions (Sundar and Chakravarty, 2015). The macular type features hypopigmented flat patches, the papular form presents with small raised lesions often hypopigmented, and the nodular type consists of larger nodules that may ulcerate. A mixed form displays both papular and nodular characteristics. These clinical variations complicate accurate diagnosis as they resemble other skin disorders like leprosy, vitiligo, or psoriasis. Post apparent cure of VL, the parasite persists in these lesions, acting as a reservoir of infection, posing a significant epidemiological threat to VL elimination efforts in endemic areas (Mondal et al., 2019; Singh et al., 2021). Hence, PKDL act as an intermediate pathological state observed after VL treatment, characterized by significant geographical variation in its presentation due to host immune responses, drug regimens, and environmental factors. In Africa, over 50% of treated VL patients develop PKDL within 6–12 months, typically showing nodular or papular lesions. In contrast, the incidence of PKDL in the ISC ranges from 5 to 20%, typically occurring within 3 years post-treatment with macular rashes being prevalent, associated with a lower parasite burden than polymorphic lesions (Routaray et al., 2022). Interestingly, PKDL cases have been documented even in Bangladesh among patients without prior VL history (Mondal et al., 2010). Several studies have reported that PKDL patients with skin lesions harbour parasites in internal organs such as the bone marrow, lymph nodes, or spleen. This condition is termed para-kala-azar dermal leishmaniasis (para-KDL). Earlier considered rare in the ISC, recent studies from Bihar, India, have identified at least nine para-KDL cases, underscoring a possible overlap between visceral and dermal form of the disease (Kumar et al., 2016). The rising co-occurrence of VL alongside PKDL highlights the urgent need for improved understanding of disease pathology, early diagnosis, and optimized treatment strategies.

Multiple factors are implicated in PKDL pathogenesis, one of which is the chemotherapeutic regimen used during VL treatment. Patients undergoing therapy with SAG (Sodium stibogluconate) demonstrate a significant likelihood of developing PKDL, while those treated with miltefosine and AmBisome have also been shown to develop the disease (Croft, 2008). Thus, SSG treatment as a sole reason for PKDL development cannot be established (Mondal and Khan, 2011; Thakur et al., 2008). Initially about 27% of PKDL cases were misdiagnosed at primary health centers (PHC) have raised serious concerns. Leprosy was misdiagnosed in maximum cases, lack of interest in patients to seek professional help has worsened the scenario. Only 25% of patients approach the clinicians for treatment due to serious cosmetic disfigurement (Ramesh et al., 2015). The rest of the patients surviving with lesions continue to be potential anthroponotic reservoirs for *Leishmania donovani* parasite (Singh et al., 2021; Inbar et al., 2016; Al-Salem et al., 2016). To overcome these hurdles, the first priority lies in correct diagnosis of PKDL at remote locations which will in-turn reduce the tie gap between onset of infection and seeking of healthcare by the patients.

## 2 Diagnosis of PKDL

The diagnostic approach of PKDL currently relies on clinical examination, individuals past history of VL, nature of skin lesion, and other dermal manifestations. Detection of LD bodies in skin biopsy samples, slit skin aspirate or bone marrow aspirate under the microscopy remains the gold standard for PKDL diagnosis. However, this method is tedious and its accuracy is limited, particularly in macular lesions where the parasite load is low, leading to potentially false-negative results. Detection accuracy ranges 67–100% for nodular lesions, whereas 36–69% for papular lesions and only 7–33% for macular forms (Verma et al., 2013; Dixit et al., 2020). Immunological studies help to locate antibodies in blood through techniques like Direct Agglutination test (DAT) or rK39 antigen dipstick methods. Although these tests are valuable for initial screening, they cannot distinguish between active and past infections, as antibodies may persist long after VL cure. Non-invasive diagnostic approaches, including enzyme-linked immunosorbent assay (ELISA) and urine-based dipstick tests, have also shown promising results for PKDL detection (Sundar and Rai, 2002; Sundar et al., 1998; Zijlstra et al., 2001). Non-invasive techniques to detect PKDL from urine samples using ELISA and dipstick methods have been developed with good outcomes (Ejazi et al., 2016; Ejazi et al., 2018). Polymerase Chain Reaction (PCR) targeting *Leishmania*-specific DNA sequences has emerged as a pivotal method due to its high reliability and specificity. Molecular biomarkers are gaining attention in detection of PKDL due to their reliable and specific results (Singh et al., 2005). Conventional methods might not be able to detect asymptomatic cases or cases where parasite burden is negligible (Lachaud et al., 2017). Better methods of detection have been developed using the basic principle of PCR. Nested PCR uses two sets of primers in two subsequent reactions. This increases the efficiency of detection of parasite (Noyes et al., 1998). Multiplex PCR gives an advantage of detecting more than one species of the parasite in a single reaction mixture using multiple sets of primers (Harris et al., 1998; Conter et al., 2018). Quantitative PCR (qPCR) helps to quantitatively determine parasite load using SYBR green or TaqMan methods in real-time (Mehrotra et al., 2025; Galluzzi et al., 2018; Kumar et al., 2024). The conserved 18S rRNA region is frequently used as a genetic target for *Leishmania* detection (Schulz et al., 2003). Using Bst DNA polymerase, a new technique has been developed to amplify DNA under isothermal condition known as Loop-mediated isothermal amplification (LAMP). This method has shown good performance in diagnosis of both VL and PKDL (Dixit et al., 2021; Nzelu et al., 2019).

**Figure 1:**
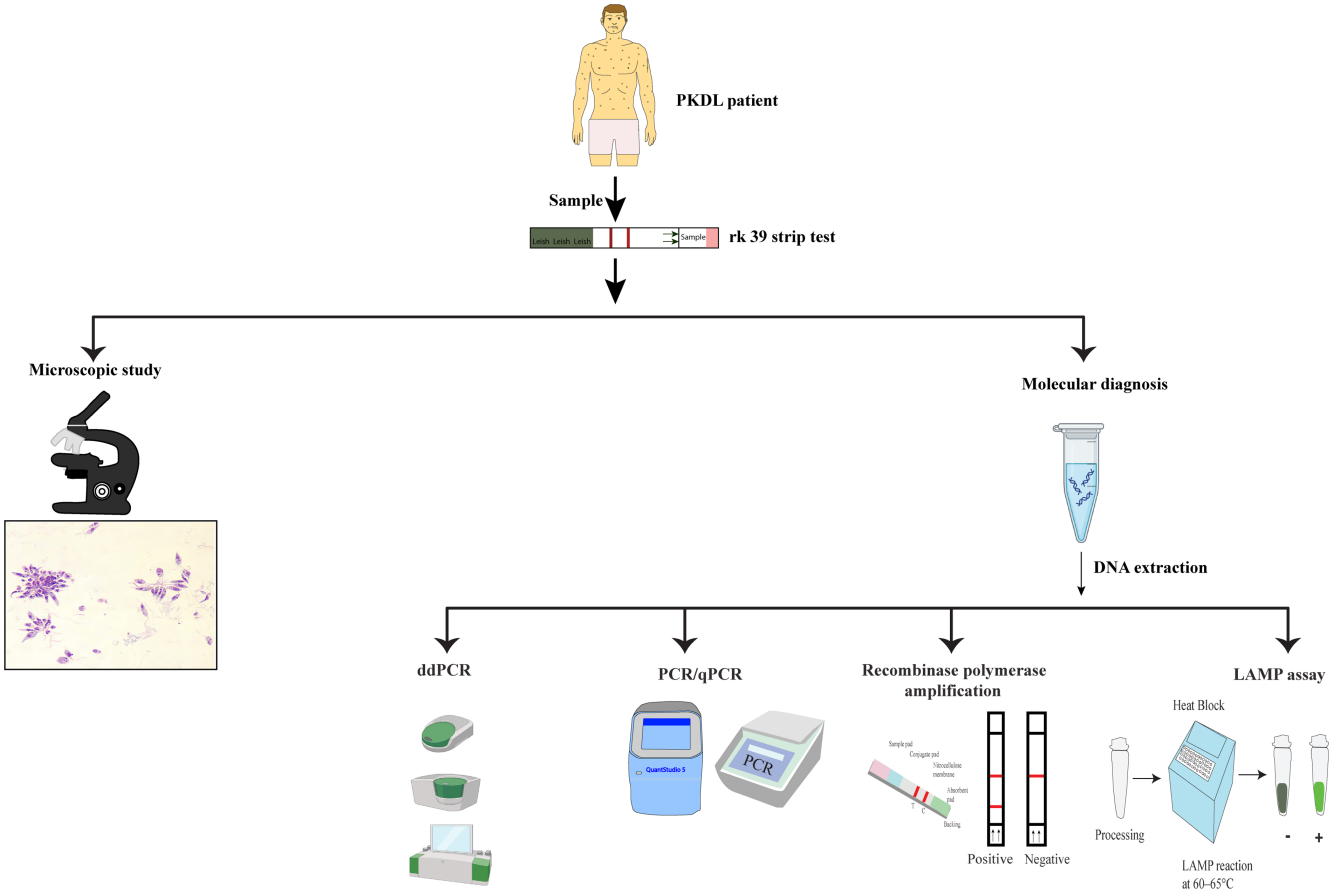
Schematic representation of the diagnostic approach for PKDL: a sample from a suspected PKDL patient is tested using the rK39 strip test. The diagnostic workflow is divided into three main pathways: (i) Microscopic study: direct visualization of *Leishmania* parasites under a microscope; (ii) molecular diagnosis by polymerase chain reaction (PCR/qPCR), recombinase polymerase amplification (RPA), and loop-mediated isothermal amplification (LAMP) assay. These molecular methods provide sensitive detection of *Leishmania* DNA to confirm the diagnosis of PKDL.

Isothermal amplification of DNA at low temperature is also used in Recombinase Polymerase Assay (RPA) to detect parasites. Temperatures as low as 42 °C is enough to run the reaction which can be even provided by body heat. Results are obtained very fast since the reaction time is of only 15 min. The technique becomes more suitable for remote field settings as it does not require any thermal cycler machine or sophisticated laboratory arrangements. RPA has been shown to have 100% specificity and 96% sensitivity in VL diagnosis. Not only for VL but also for PKDL, RPA has been proved to be 91.3% specific and 100% sensitive. More studies need to be conducted to establish RPA as a promising and reliable diagnostic approach for PKDL (Chowdhury et al., 2020; Roy et al., 2023; Figure 1; Table 1).

**Table 1:** Different diagnostic tests involved in the leishmaniasis with their targets [adapted from Mehrotra et al., 2025].

Methods of Diagnosis	Sensitivity	Specificity	Targets	Advantages	Disadvantages
1. Microscopy	93–99% (splenic aspirate), 60–80% (bone marrow aspirate)	100%	Phenotypic morphology	Simple, low cost, widely available	Requires expertise, invasive sample collection (splenic, bone marrow aspirates), low sensitivity in peripheral blood
2. IHC	50–85%	90–100%	DNA	Useful for detecting amastigotes in tissue samples	Requires specialized reagents and equipment, labor-intensive
3. CISH	55–75%	100%	DNA	No need for specialized fluorescence microscopy, unlike FISH	Requires extensive sample preparation, Sensitivity can be lower compared to FISH
4. FISH	50–80%	93–100%	DNA	Allows visualization of parasite DNA within tissue	Expensive, requires fluorescence microscopy, not routinely available
5. ddPCR	100%	80%	DNA	Provides higher analytical sensitivity, precision, and robustness against PCR inhibitors	High operational costs, restricted multiplexing capacity, and an even more complex workflow.
6. rK39 dip stick	100% (Indian subcontinent), 67–71% (outside the subcontinent)	88–100%	Immunoglobulin	Rapid, non-invasive, easy to use in the field	Variability in sensitivity outside Indian subcontinent
7. DAT	91–100%	72–95%	Immunoglobulin	Simple, low-cost	False positives possible, lower specificity compared to molecular tests
8. Conventional PCR	93–99% (splenic aspirate), 53–86% (bone marrow aspirate), and 53–65% (lymph node aspirate) 62–93.7% (peripheral blood),	100%	DNA	High sensitivity in invasive samples (e.g., splenic aspirates)	Invasive sampling required for best sensitivity, lower sensitivity in blood
9. Nested PCR	100%	100%	DNA	Extremely high sensitivity and specificity	Time-consuming, higher risk of contamination
10. qPCR	91–100%	29–100%	DNA	Quantitative results, sensitive	Variability in specificity, requires expertise and expensive equipment
11. LAMP	80–100%	94–100%	DNA	Simple, does not require thermocycler, field-applicable	Limited use in routine labs, lower sensitivity in certain sample types like peripheral blood

Droplet digital PCR (ddPCR) is an advanced PCR-based technology in which a nucleic acid sample is divided into thousands of discrete nanolitre-sized droplets, with PCR amplification occurring independently within each partition. Following end-point amplification, target molecules are quantified absolutely using Poisson statistical analysis, eliminating the need for external calibration curves (Hindson et al., 2011). In leishmaniasis, ddPCR has emerged as a powerful tool for sensitive detection and accurate quantification of *Leishmania* DNA in clinical samples. Its application has been demonstrated in visceral and cutaneous leishmaniasis for diagnosis, parasite load estimation, treatment monitoring, and early detection of relapse (Ramírez et al., 2019). Unlike qPCR, ddPCR does not require standard curves and provides higher analytical sensitivity, precision, and robustness against PCR inhibitors, making it particularly suitable for detecting low-abundance targets (Tsokana et al., 2023). These advantages are especially relevant for PKDL, where parasite burden is often low, patchy, and persistent.

## 3 Treatment options for VL and PKDL

For VL, pentavalent antimonial (SbV) has been the primary treatment for decades, but issues like daily administration, diminishing efficacy, and safety concerns prompted a shift to Amphotericin B (AmB) in India due to widespread SbV resistance. Although AmB is nearly 100% effective, it presents challenges such as infusion reactions, nephrotoxicity, and extensive monitoring requirements (Roatt et al., 2020). Consequently, the Indian control program transitioned to oral miltefosine for convenience, but complications like procurement issues, teratogenicity, and long treatment duration led to the adoption of single-dose AmBisome as the current first-line treatment (Sundar et al., 2010).

In case of PKDL, once diagnosed, the next approach is to choose the correct drug for treatment. Variability in disease outcomes makes the treatment regime to be complex and challenging. Severe cases of PKDL in the East African population are treated with SSG or with AmBisome (Zijlstra et al., 2003; Zijlstra, 2016). Miltefosine, remains the first drug of choice for clinicians treating PKDL in India. Miltefosine was administered orally at a dose of 2.5 mg/kg/day for 12 weeks. Patients weighing more than 25 kg were given 100 mg daily, with one 50 mg capsule taken with meals in the morning and evening. One 50 mg capsule was administered daily to patients weighing less than 25 kg (Ramesh et al., 2015; Sundar et al., 2015). Decline in efficacy of miltefosine treatment has been noted in the past few years. Gastrointestinal upset and ophthalmic adversities are seen in patients on prolonged treatment of miltefosine. This often leads to discontinuity of the medicine and incomplete cure of the disease (Ghosh et al., 2015; Pal et al., 2024). Though nephrotoxicity is a serious concern, 1 mg/kg of amphotericin B has been administered to patients in the form of 60–80 infusions for over 4 months, often due to such long duration of treatment, patients become reluctant to medication (Rabi Das et al., 2017). AmBisome, when administered to patients of Bangladesh, has shown cure in 78% cases with no toxicity, 15 mg/kg is divided into 3 mg/kg bi-weekly and is being prescribed to patients for 3 weeks (den Boer et al., 2018).

When paromomycin, an aminoglycosidic antibiotic, was tried to treat patients with PKDL, the outcomes were not satisfactory (Sundar et al., 2014).

Since the 1920s, SSG has been the primary drug for treating leishmaniasis. However, its use is often limited by serious adverse effects, most notably cardiac arrhythmias and acute pancreatitis, which in some cases may be life-threatening (Musa et al., 2012; Cesur et al., 2002). Though SSG was the first drug of choice, rising cases of antimony resistance paired with severe toxic side effects in patients have caused discontinuity of its use (Choudhury et al., 2008). In the past 10 years, the reliance on SSG in the ISC has declined significantly due to the emergence of extensive drug resistance (Vanaerschot et al., 2012). Despite Rifampin being used to treat leishmaniasis, no such effective study has been conducted in PKDL (Tsankov and Angelova, 2003; Kumari et al., 2024). Miltefosine and AmBisome are used in a combination nowadays and have shown better results with decreased toxicity. Despite the efficacy of above-mentioned drugs, often the outcomes vary depending on lesion types in PKDL. Gradual decrease in parasite load is seen in macular lesions as compared to polymorphic lesions, when AmBisome is used as drug of choice (Kumar et al., 2023). These complex outcomes depending on heterogeneity of lesions shifts the focus to investigate the molecular and immunological mechanisms to decode the parasite survival strategies. List of the drugs and combination of drugs used in the treatment of leishmaniasis are given in the Table 2.

**Table 2:** Different drugs or drug combination used in the treatment of leishmaniasis [Adapted from (Sundar et al., 2024)].

S. no.	Drugs/Drug combination	Disease	Dosing duration	References
1	Amphotericin B (AmB) deoxycholate	VL	5–6 weeks	Chakravarty and Sundar (2010) and Roatt et al. (2020)
2	Oral miltefosine	VL	28 days	Roatt et al. (2020)
3	liposomal amphotericin B (L-AmB)	VL	Single dose (10 mg/Kg)	Sundar et al. (2010)
4	Multidrug Therapies (Phase II trials)			
	Sb^V^ (Intravascular or Intramuscular) and Intramuscular paromomycin (PM)	VL	17 days	Musa et al. (2012)
	LAmB (5 mg/mL) + Miltefosine	VL	7 days	Sundar et al. (2010)
	LAmB (5 mg/mL) + Miltefosine	VL	10 days	
	LAmB (5 mg/ml) + Miltefosine	VL	14 days	
5	Multidrug Therapies (Phase III trials)			
	LAmB (5 mg/ml) + 50 mg Oral Miltefosine (Phase III trials)	VL	7 days	Sundar et al. (2011)
	LAmB (5 mg/mL) + 11 mg/kg Intramuscular paromomycin (Phase III trials)	VL	10 days	
	Oral miltefosine (10 days) + 11 mg/kg Intramuscular paromomycin (Phase III trials)	VL	10 days	
6	Sodium stibogluconate (SSG) 20 mg/kg	CL	20–28 days	Navin et al. (1992), Arevalo et al. (2007), and Romero et al. (2001)
7	Miltefosine at 1.5–2.5 mg/kg/day	CL	28 days	Chrusciak-Talhari et al. (2011) and Machado et al. (2010)
8	LAmB at 3 mg/kg	CL	5 days	Ono et al. (2011)
9	12-week Miltefosine + 70–80 dose of AmB	PKDL	4 months	Chakravarty and Sundar (2019)
10	SSG at 20 mg/kg/day per day	PKDL	2 months	

## 4 Mechanisms underlying the progression of VL to PKDL

**Figure 2:**
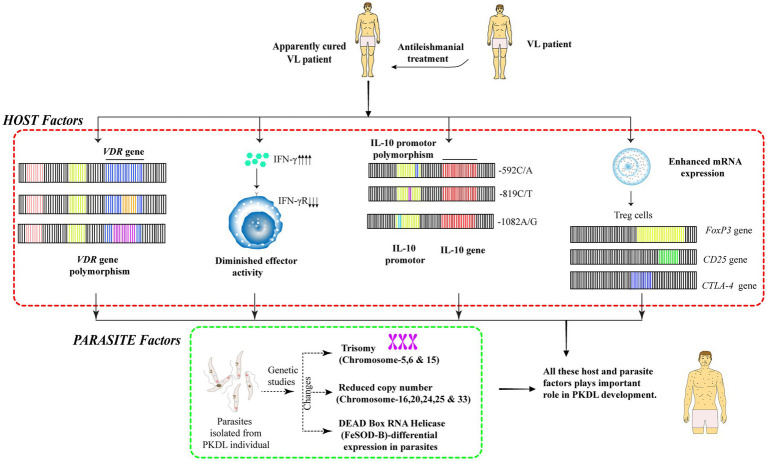
Host and parasite genetic factors contributing to PKDL development after VL treatment. Genetic factors contribute to PKDL development in a minority of patients cured from VL, involving both host and parasite-related genetic changes. Host factors include polymorphisms in genes regulating immune responses and metabolism. Parasite isolated from PKDL patients show notable genetic differences from those found in VL alone.

PKDL development represents a complex immune reconstitution inflammatory syndrome (IRIS) following apparently successful VL treatment (Chatterjee et al., 2020; Khalil et al., 2013). Inadequate pharmacokinetic and pharmacodynamic drug exposure is a major determinant of treatment failure. Sub-optimal absorption, insufficient tissue penetration, poor treatment adherence, vomiting, and shortened regimens, can all result in drug concentrations that are inadequate to clear intracellular parasites from reticuloendothelial organs (Sundar and Chakravarty, 2015). Such subtherapeutic exposure may not lead to immediate clinical failure but allows residual parasites to survive, predisposing patients to relapse months after apparent cure (Dorlo et al., 2017). High baseline parasite burden, delayed treatment initiation, and younger age have also been consistently associated with higher relapse and PKDL risk, indicating that disease severity and host immune maturity play important roles in long-term outcomes (Goyal et al., 2020). During active VL, patients exhibit profound immunosuppression characterized by suppressed cellular immunity, elevated IL-10 and TGF-*β* levels, and impaired T-cell responses (Volpedo et al., 2021; Gasim et al., 2000). Following antileishmanial therapy, immune reconstitution occurs with restoration of Th-1 responses, increased IFN-*γ* production, and decreased regulatory cytokine levels (Chatterjee et al., 2020; Volpedo et al., 2021). However, PKDL pathogenesis involves a unique dissociation of immune responses between systemic and dermal compartments (Zijlstra, 2016; Figure 2)). While systemic immunity is restored with persistent Th-1 responses and IFN-γ production, the skin maintains an immunosuppressive environment with persistent IL-10 production (Zijlstra, 2016). This creates conditions favorable for parasite persistence in dermal tissues, where *Leishmania* parasites which escaped VL treatment can proliferate and trigger inflammatory responses (Volpedo et al., 2021; Gasim et al., 2000).

The immune reconstitution process leads to the activation and dermal homing of *Leishmania* reactive T cells from peripheral blood (Volpedo et al., 2021; Gasim et al., 2000). These cells infiltrate cutaneous tissues and produce inflammatory mediators, particularly IFN-*γ*, which exacerbates the dermal inflammatory response, characteristic of PKDL (Volpedo et al., 2021). The resulting immune response demonstrates features of both Th-1 and Th-2 patterns, with IL-10 persistence in skin lesions despite systemic Th-1 dominance (Zijlstra, 2016; Chatterjee et al., 2020). Central to PKDL pathogenesis is the persistence of *Leishmania* parasites in skin tissues following VL treatment (Volpedo et al., 2021; Arumugam et al., 2022). Parasites spread to dermal tissues during the acute phase of VL and can survive antileishmanial therapy in these sites (Sundar and Chakravarty, 2015). The skin provides a niche where parasites may persist in low numbers, protected from complete drug medication and immune clearance (Arumugam et al., 2022). Parasite persistence is facilitated by the unique dermal immune environment, characterized by alternatively activated M2 macrophages that suppress cell-mediated immunity and promote parasite survival (Zijlstra, 2016). Elevated levels of IL10, TGF*β*, and regulatory T cells (Treg) in skin lesions create an immunosuppressive milieu that allows parasite multiplication (Zijlstra, 2016; Chatterjee et al., 2020). The ratio of inflammatory (TNF-*α*) to anti-inflammatory (IL-10) cytokines in PKDL skin biopsies is 2.66, indicating a mixed inflammatory response that fails to achieve complete parasite clearance (Zijlstra, 2016).

Autophagy-regulated through nutrient and stress sensors such as PI3K-Akt–mTOR (autophagy inhibition) and AMPK-ULK1 (autophagy induction), is a central pathway for intracellular pathogen restriction and antigen processing, but can be functionally suppressed to permit parasite persistence (Shoeran and Anand, 2025). In parallel, macrophage polarization is controlled by cytokine-driven signaling: IFN-γ/JAK-STAT1 promotes iNOS-dependent microbicidal activity and Th1-supportive functions, whereas IL-4/IL-13/JAK–STAT6 and IL-10/JAK-STAT3 favor regulatory/tissue repair programs and reduce leishmanicidal capacity, enabling residual parasites to survive after apparent VL cure (Zijlstra, 2016; Mukhopadhyay et al., 2014). PKDL is therefore consistent with a post-treatment state of immune compartmentalization, where systemic control improves but cutaneous macrophage signaling (including sustained STAT3-linked regulation and impaired autophagy flux) allows parasite persistence in the skin and subsequent dermal lesion development (Mukhopadhyay et al., 2014).

Ultraviolet (UV) light exposure has gained recognition as a significant environmental determinant in the pathogenesis of PKDL. The clustering of PKDL lesions predominantly on sun-exposed regions such as the face, neck, and arms supports the hypothesis that chronic UV-B radiation plays a key role in disease initiation and progression by inducing localized immunosuppression (Mukhopadhyay et al., 2014; Ismail et al., 2006). UV-B exposure disturbs cutaneous immune homeostasis by impairing the function and morphology of epidermal Langerhans cells (E-LCs), a specialized antigen-presenting cells (APCs) essential for initiating Th-1 immune responses and by altering the cytokine milieu within the skin microenvironment Ultraviolet light induced injury: immunological and inflammatory effects (Amerio et al., 2009). Experimental and clinical studies demonstrate that UV-B irradiation reduces E-LC denticity, downregulates HLA-DR and co-stimulatory molecules such as CD80/CD86, and simultaneously elevates interleukin-10 (IL-10) and transforming growth factor-β (TGF-β), collectively dampening effective antigen presentation (Gedda et al., 2020). Apart from these, heterogeneity is seen in clinical outcome of PKDL. The complex role of immune machinery is very less understood. Multiple histological and molecular investigations demonstrate that macular PKDL lesions typically contain significantly lower *Leishmania donovani* parasite loads than papulo-nodular/polymorphic disease, frequently falling below the sensitivity of conventional slit-skin smear microscopy and becoming reliably detectable only by high-sensitivity molecular methods such as qPCR (Ghosh et al., 2018; Sengupta et al., 2019). This parasite-poor phenotype correlates with a sparse, superficial, and patchy dermal infiltrate, indicating limited recruitment of inflammatory and effector immune cells into the skin compared with nodular forms (Mukhopadhyay et al., 2014). Immunophenotyping further supports these differences: macular PKDL exhibits reduced CD68^+^ macrophage density, which likely restricts intracellular niches required for parasite persistence and replication, and is accompanied by lower CD8^+^ T-cell infiltration than nodular/polymorphic lesions (Moulik et al., 2018). Macular PKDL is characterized by significant downregulation of HLA-DR expression in dermal antigen-presenting cells, leading to weakened local antigen presentation and reduced effector immune responses. This indicates a state of partial immune containment that suppresses severe dermal parasitism and inflammation, but fails to eliminate residual parasites, resulting in lasting lesions with low parasite density (Mukhopadhyay et al., 2014; Zijlstra, 2019).

In contrast, polymorphic/nodular PKDL is characterized by dense dermal inflammation, prominent macrophage and CD8^+^ T-cell enrichment, and significantly higher parasite loads relative to macular disease (Sengupta et al., 2019). This passage discusses the coexistence of inflammatory responses with parasite persistence, highlighting the role of immunoregulatory pathways, particularly IL-10, in suppressing leishmanicidal mechanisms of macrophages. It suggests that the morphology of PKDL lesions reflects a balance between local parasite presence, permissive host cells, and the interactions between effector and regulatory immune responses in the skin environment (Mukhopadhyay et al., 2014; Zijlstra, 2019).

## 5 Genetic factors involved in PKDL development

The development of PKDL is believed to result primarily from a combination of factors, including immunosuppression, the reactivation of residual *Leishmania* parasites, or reinfection in individuals who have previously acquired visceral immunity. Despite significant research, the precise mechanisms that allow the parasite to persist and subsequently manifest as dermal lesions are not yet fully elucidated. Current evidence increasingly points toward a multifactorial basis, where both host-related determinants-such as altered immune regulation, genetic susceptibility, and cytokine imbalances-as well as parasite-associated traits-such as antigenic variation, adaptive stress responses, and drug tolerance-play pivotal roles. These interactions are thought to create a permissive environment that enables the parasite to evade immune clearance, survive within host tissues, and ultimately drive the chronic cutaneous pathology characteristic of PKDL (Foote and Handman, 2005; Blackwell, 1996).

### 5.1 Host specific genetic factors

The progression of the disease is influenced by genetic variations in host genes encoding heat shock proteins and solute carriers. Furthermore, immune-regulatory gene expression is modulated by epigenetic mechanisms such as DNA methylation and histone acetylation (Afrin et al., 2019). Epigenetic silencing of key cytokine signaling pathways may impair parasite clearance and contribute to disease persistence. The interferon-gamma receptor 1 (*IFNGR1*) gene represents one of the most significant genetic determinants of PKDL susceptibility. Polymorphisms in the promoter region of *IFNGR1* have been consistently associated with increased risk of developing PKDL following successful treatment of VL. Studies in Sudanese populations demonstrate that specific haplotypes comprising four promoter polymorphisms (−470 ins/delTT, −270 T/C, −56 T/C, and +95 T/C) show significant global association with PKDL development. These genetic variants affect *IFNGR1* expression levels, leading to reduced responsiveness to IFN-*γ* despite elevated cytokine production. PKDL patients demonstrate significantly lower *IFNGR1* expression at both mRNA and protein levels compared to healthy controls, explaining the paradox of high IFN-γ levels but poor parasite clearance. The restoration of IFNGR1 expression following successful treatment correlates with parasite elimination and clinical improvement (Mohamed et al., 2003; Salih et al., 2007; Ansari et al., 2006; Salih et al., 2014).

Experimental evidence suggests that IL-10 serves as a key immunoregulatory factor in the progression of VL. In PKDL, IL-10 has been reported as the dominant cytokine within skin lesions, with elevated concentrations also detected in patient plasma. Nonetheless, IFN-γ is consistently present in lesions [64] and has additionally been localized to keratinocytes and sweat glands in affected individuals. Peripheral blood mononuclear cells (PBMCs) from Sudanese PKDL patients, when stimulated with *Leishmania* antigens, typically exhibit both proliferative responses and secretion of IFN-γ alongside IL-10. Silva et al. (1992) proposed that IL-10 within dermal lesions does not suppress IFN-γ production directly but may instead interfere with its effector activity. Interleukin-10 (*IL10*) gene variants significantly influence PKDL pathogenesis through their effects on immune regulation and parasite persistence. Three key promoter polymorphisms (−1082A/G, -819C/T, and -592C/A) have been extensively studied in relation to PKDL susceptibility. The functional significance of these polymorphisms lies in their ability to modulate IL-10 production levels. The -592C/A polymorphism is associated with diminished IL-10 production, while the -1082A/G variant affects transcriptional activity. The GCC and ATA haplotypes, which are common in PKDL-endemic populations, are associated with enhanced IL-10 secretion and may contribute to the immunosuppressive environment that facilitates parasite persistence. While single-point analyses show limited associations, haplotype analysis reveals borderline significance for the AA haplotype across -592C/A and -1082A/G markers (Silva et al., 1992; Hollander, 2012; Farouk et al., 2010).

Genetic factors affecting Treg function significantly influence PKDL development. Polymorphisms in genes encoding FoxP3, CD25, and CTLA-4 affect Treg cell accumulation and function in skin lesions. Elevated mRNA levels of these Treg markers correlate directly with parasitic load in PKDL patients, indicating that genetic variants affecting Treg responses may determine disease severity and duration (Figure 2).

**Figure 3:**
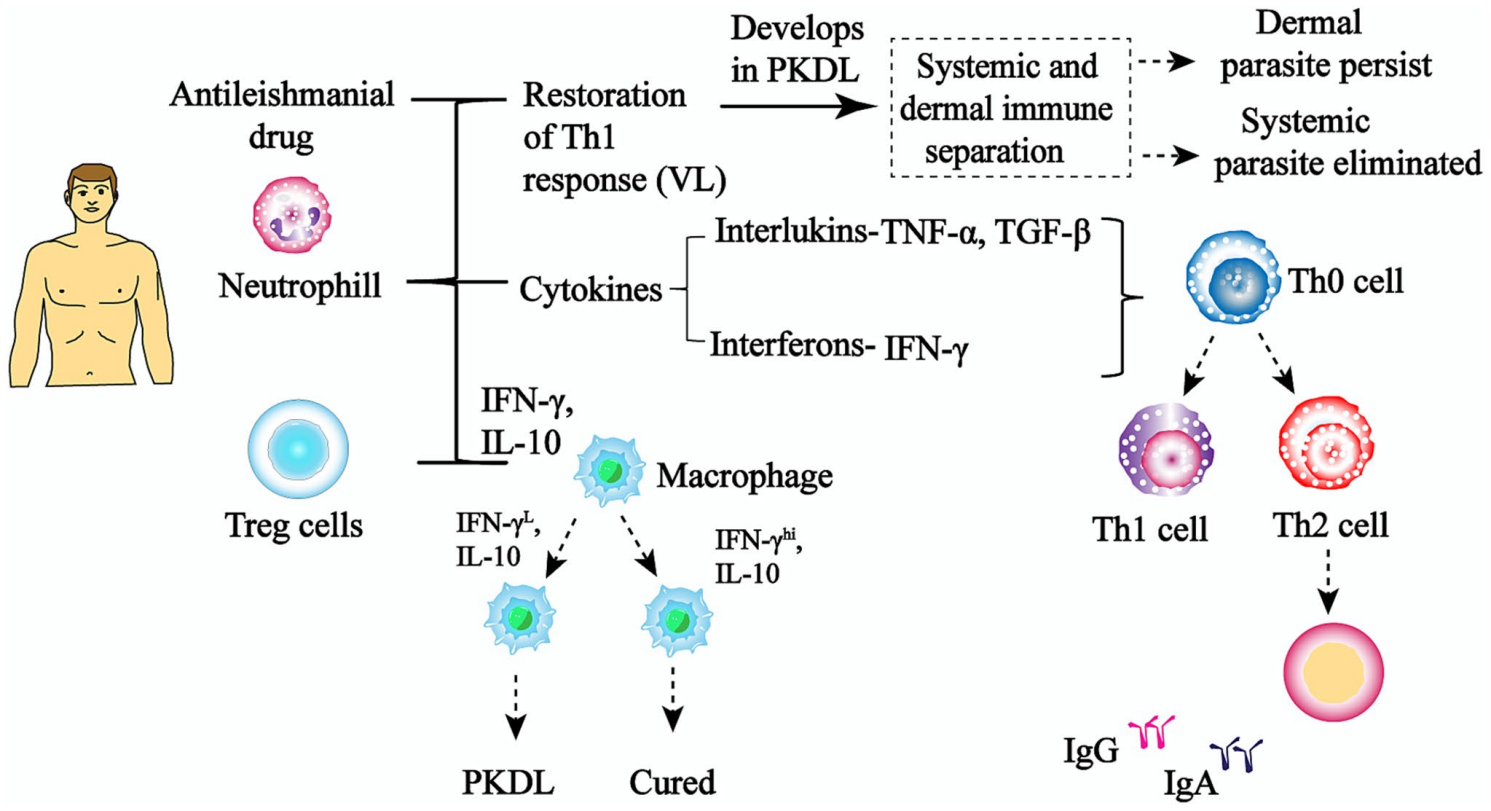
Specific immunological response in VL and PKDL. The picture shows how the immune system changes after therapy for VL. It reveals that those who have healed have their Th1-type immunity restored, macrophages activated by IFN-γ, and parasites are eliminated. On the other hand, an alternate pathway shows immunological dysregulation, as shown by the activity of regulatory T-cells, the synthesis of IL-10, the failure to clear parasites, and the parasites persistence in the skin. This can cause post-kala-azar dermal leishmaniasis even if the disease seems to be cured.

One of the main indications of PKDL is altered vitamin D signaling. For example, there are increased levels of 1α,25-dihydroxyvitamin D3 (1,25-D3) in the blood and vitamin D3-associated genes are upregulated. These genes include VDR (which is responsible for nuclear signaling of 1,25-D3), CYP27B1 (which encodes vitamin D-1α-hydroxylase, which turns inactive prohormone into its bioactive 1,25-D3 form), and LL-37 (a downstream antimicrobial effector peptide cathelecidin of the vitamin D signaling pathway). This is another sign of M2 polarization (Mukhopadhyay et al., 2015; Das et al., 2017). A recent study has investigated the role of Vitamin D receptor (*VDR*) gene polymorphism and vitamin D status as risk factors for VL and PKDL among patients in Bihar, India-a major endemic region. Researchers analyzed polymorphisms in the *VDR* gene, particularly *BsmI* (rs1544410), *ApaI* (rs7975232), and *TaqI* (rs731236), in VL and PKDL patients compared to healthy controls (Lemos et al., 2008). The AA and GA genotypes of the *BsmI* gene polymorphism were found to be significantly associated with increased risk of both VL and PKDL (odds ratios for AA: 7.03 in VL, 4.98 in PKDL; for GA: 2.49 in VL, 2.97 in PKDL). Serum vitamin D levels were much lower in VL patients (22.41 ± 10.57 ng/mL) than in PKDL patients (42.19 ± 10.84 ng/mL), indicating a possible relationship between vitamin D deficiency and VL susceptibility. Additionally, gene expression analysis revealed altered VDR and CYP27B1 profiles, with upregulation in PKDL and downregulation in VL, suggesting differential vitamin D pathway regulation in the two diseases. The findings highlight *BsmI* polymorphism as a genetic risk factor and underscore the importance of adequate vitamin D status, particularly in nutritionally compromised populations in leishmaniasis associated endemic areas (Kalim et al., 2025; Figure 3).

### 5.2 Parasite specific genetic factors

*Leishmania* parasite exhibits epigenetic plasticity, enabling dynamic modulation of its gene expression to adapt to immune pressure and therapeutic interventions. Additionally, environmental factors including co-infections, poor living conditions, and malnutrition interact with host genetic predispositions to further dysregulate immune responses and exacerbate disease progression (Sudarshan and Sharan, 2025).

A total of 138 transfer RNA (tRNA) genes were identified in the genome of the isolate, which is substantially higher than the number reported in other sequenced *Leishmania* species [1]. Sequence mapping revealed that 4.2% of reads aligned with *Leishmania infantum*, 4.6% with *Leishmania major*, and 1.8% with *Leishmania braziliensis* (Gupta et al., 2015). Comparative genomic analysis between the PKDL isolate and Indian *Leishmania donovani* strains showed a high degree of sequence conservation, with 98.3% genome coverage relative to strain Ld2001, 88.2% to Ld39, and 70.3% to BHU1095, resulting in an overall nucleotide homology of 98.0% among Indian isolates (Gupta et al., 2015). BLAST analysis further identified 11,281 sequence reads corresponding to *Leptomonas seymouri*, indicating a possible co-infection, consistent with previous reports of *Leptomonas* co-existence in Indian VL and PKDL cases (Ghosh et al., 2012; Singh et al., 2013). In addition, 893 contigs showed similarity to the heterotrophic bacterium *Parvibaculum lavamentivorans* DS-1, covering approximately 53.8% of its genome (Schleheck et al., 2011). Together, these findings suggest the presence of superinfection or microbial association within the PKDL isolate, which may influence parasite biology and disease complexity (Alves et al., 2013).

Differential gene expression between two stages have been studied through molecular techniques like arbitrary primed PCR (AP PCR), cDNA micro array and genomic microarray. Upregulation of cell surface proteins have been seen in parasites isolated from PKDL patients which were absent in VL isolates. LdP13 and *β*-tubulin loci have been distinct in VL and PKDL isolates (Ansari et al., 2006; Salih et al., 2014). AP PCR analysis of 14 dermal lesion isolates from PKDL and 3 bone marrow-derived VL isolates revealed differential gene expression in putative phosphodiesterase, DEAD BOX RNA helicase, and iron superoxide dismutase b (FeSODB). While the expression patterns were similar between PKDL and VL isolates, polymorphic fragments linked to these genes were more pronounced in PKDL, indicating important stage-regulated molecular adaptations for parasite survival in different environments. FeSODB assists the parasite in coping with oxidative stress, enhancing its survival in challenging conditions (Gupta et al., 2015; Figure 3). The adaptation of parasites to skin tissues involves crucial chromosomal changes that enhance survival. Whole-genome sequencing has revealed unique aneuploidy patterns in *L. donovani* strains from PKDL patients, with specific chromosomal copy number alterations compared to strains causing VL. Analysis shows trisomy in chromosomes 5, 6, and 15 in para-KDL strains, alongside reduced numbers in chromosomes 16, 20, 24, 25, and 33. These chromosomal alterations are important as they harbour genes for ATP-binding cassette (ABC) transporters and metabolic enzymes necessary for the parasite’s survival in various tissue environments (Sarraf et al., 2021).

Transcriptome analysis reveals significant differences between PKDL and VL dermal fibroblasts. In PKDL, key hub genes such as MMP2, IL1*β*, CXCL8, IFIH1, NFKB1A, IL6, ISG15, and EGFR are downregulated, while ACTB, HSP90AA1, RAB7A, and RPS27A are upregulated. PKDL fibroblasts enhance antigen presentation via MHC class I, promoting CD8^+^ T-cell responses. In contrast, VL fibroblasts are characterized by increased NF-κβ-driven chemokines, potentially attracting NK cells and monocytes through CD4^+^ T-cell immunity (Singh et al., 2023).

Antimonials such as SSG were once widely used, and their declining efficacy has been associated with parasite adaptations. A key player is the aquaglyceroporin 1 (*AQP1*) gene of *Leishmania donovani*, which encodes a membrane channel responsible for transporting trivalent antimony (SbIII), the active form of the drug. Clinical studies assessing Indian isolates demonstrated that while AQP1 is not deleted or structurally altered in resistant strains, its expression is markedly reduced compared with susceptible isolates. This downregulation leads to impaired SbIII uptake, thereby diminishing drug efficacy. Functional assays restoring *AQP1* expression partially reversed resistance, confirming its central role in antimony susceptibility. In the context of PKDL, where residual parasites persist after VL treatment and may harbour resistance traits, reduced *AQP1* expression could critically influence therapeutic outcomes. These findings suggest that monitoring *AQP1* status in dermal isolates may provide valuable insights into treatment response in PKDL. Furthermore, *AQP1* represents not only a molecular marker for resistance surveillance but also a potential target to overcome therapeutic challenges in PKDL management (Mandal et al., 2010).

## 6 Discussion

PKDL reflects the complex biological and epidemiological challenges that hinder VL elimination. Although VL therapy restores systemic Th1 immunity, parasites often persist in dermal tissues where an IL 10 dominant, UV modulated microenvironment enables survival. This compartmentalized immune response systemic Th1 activation but local immunosuppression forms the basis of PKDL pathogenesis. Host genetic factors, including polymorphisms in IFNGR1, IL10, VDR, and genes regulating Treg function, further modulate cytokine responses and influence susceptibility, while parasite-specific adaptations such as aneuploidy, altered stress-response pathways, and reduced AQP1 expression reinforce persistence and drug tolerance.

The immunopathogenesis of PKDL highlights a post-treatment phase during which host-directed immunotherapy may enhance antiparasitic medications to strengthen cure and prevent recurrence or dermatological conditions. Immunotherapy should aim to restore effective leishmanicidal immunity and prevent exacerbation of inflammation, given that PKDL impairs immune function in specific body regions. Strategies that enhance Th1-mediated macrophage activation, restore autophagy flux, and readjust macrophage polarization from IL-10/STAT3–dominant regulatory states may facilitate parasite eradication in skin tissues. Another reasonable option, especially for people with skin lesions that will not go away, is to change the levels of regulatory cytokines like IL-10 or TGF-β in a way that is limited in time and takes into account the tissue.

Additionally, therapeutic vaccination or immunological enhancement following chemotherapy may enhance persistent antigen-specific memory responses and reduce the risk of relapse or PKDL by promoting immune consolidation after parasite debulking. Recent findings about T-cell dysfunction associated with checkpoints and challenges in antigen presentation within PKDL lesions suggest the potential for meticulously engineered immune checkpoint or antigen-presenting cell-targeted therapies. Still, safety concerns mean that these treatments need to be tested in full. In general, combining immunotherapy with standard treatment, based on signs of immune reconstitution and parasite persistence, is a possible way to deal with the clinical problems of relapse, PKDL, and para-PKDL that have not been solved.

Clinically heterogeneous lesions complicate early detection, and conventional microscopy remains insensitive for low-burden macular disease. Molecular diagnostics, particularly qPCR, nested PCR, LAMP and RPA, offer superior sensitivity and field compatibility, making them essential for identifying asymptomatic or atypical cases that maintain transmission. Treatment remains difficult declining miltefosine efficacy, antimonial toxicity, and variable responses to LAmB necessitate revised strategies, including shorter, combination regimens and potential host-directed therapies. Because PKDL patients act as chronic anthroponotic reservoirs, especially in the ISC and East Africa, integrating sensitive diagnostics, genomic surveillance, and improved treatment adherence is critical. A mechanistic understanding of immune reconstitution, parasite adaptation, and environmental influences will be key to interrupting transmission and achieving sustainable VL elimination.

## 7 Future perspectives

The last decade has witnessed the use of various omics studies either alone or in combination to investigate a particular anomaly in human disease. A plethora of data generated from these studies need to be integrated into statistical and arithmetical structure so that it can solve broader queries regarding the various fields of biology. The story generated till date through omics-based technologies in PKDL is incomplete and quite scattered. The application of data is limited due to lack of larger cohort of samples, regional biasness, and lack of longitudinal data follow up. PKDL continues to pose a significant challenge in the elimination of VL, acting as a reservoir for disease transmission and complicating control programs. Traditional clinical and parasitological diagnostic approaches, although useful, are often limited by poor sensitivity, delayed detection, and lack of specificity. In this context, omics-based technologies-genomics, transcriptomics, proteomics, metabolomics, and immunomics have emerged as transformative tools that provide unprecedented insights into host–parasite interactions, molecular pathogenesis, and therapeutic responses.

Genetic factors influencing innate immunity appear to play a significant role in determining susceptibility to leishmaniasis. Variants in genes regulating complement activation, such as FCN2 (encoding Ficolin-2), have been linked with a higher risk of CL (Kilpatrick and Chalmers, 2012; Assaf et al., 2012). Specifically, promoter polymorphisms that reduce FCN2 expression are associated with increased disease susceptibility (Assaf et al., 2012). Similarly, mannose-binding lectin 2 (MBL2) has been implicated in VL, where elevated serum levels and genetic polymorphisms within its promoter and exonic regions correlate with heightened vulnerability (Peres Alonso et al., 2007). Another well-established candidate, SLC11A1 (previously NRAMP1), encodes a proton/divalent cation antiporter located on phagocyte endosomal membranes. This transporter influences multiple macrophage functions, including IL-1β production, iNOS activity, MHC class II expression, TNF-*α* release, nitric oxide generation, oxidative burst, and antimicrobial responses (Blackwell et al., 2001). Functionally null alleles arising from SLC11A1 promoter or exon polymorphisms are strongly associated with increased risk of VL (Mohamed et al., 2004; Blackwell et al., 2004). In the context of PKDL, a future diagnostic method based on the CRISPR/Cas system could revolutionize early detection and management of this hidden reservoir of VL. By targeting parasite-specific genetic markers (such as kinetoplast DNA minicircles or 18 s rDNA) with Cas12a or Cas13a enzymes, the assay could deliver ultra-high sensitivity even in skin lesions with very low parasite loads, which is a current major challenge in PKDL. Coupling the CRISPR reaction to a minimal sample preparation (e.g., skin scraping or fine-needle aspirate) and isothermal amplification (such as RPA or LAMP) would allow deployment at peripheral public health centers or mobile outreach camps (Dueñas et al., 2022; Piyasiri et al., 2023). However, comparable data in PKDL remain scarce, underscoring the importance of identifying genetic susceptibility determinants in this context. Future work exploring epigenetic modifications in these candidate genes may provide crucial insights into why certain individuals progress to PKDL after VL treatment.

Research on PKDL must adopt a multidisciplinary, translational framework to close persistent gaps in pathogenesis, transmission dynamics, and clinical management. Key priorities include longitudinal studies integrating immunophenotyping, parasite genomics, and transcriptomics to define mechanisms of dermal persistence, immune dysregulation, and relapse after VL treatment, while enabling biomarker discovery for prediction of PKDL risk and therapeutic response. In parallel, molecular epidemiology using whole-genome sequencing and population genetics should clarify whether dermal parasites represent residual VL populations or skin-adapted subclones, thereby strengthening transmission models and elimination strategies. Diagnostic innovation remains critical, particularly for macular PKDL, requiring sensitive field-adapted tools such as skin swab molecular assays and host biomarker signatures to differentiate active disease from residual lesions. Therapeutically, future trials should optimize shorter, safer regimens and combination therapies, supported by pharmacovigilance and real-world effectiveness studies. A precision medicine approach, integrating parasite load, host immune signatures, and pharmacokinetic variability, could individualize treatment intensity and follow-up. Accurate, field-ready diagnostic tools are crucial for PKDL because macular lesions typically contain a low and uneven parasite load, making microscopy insensitive and increasing the risk of clinical misdiagnosis. Deployment of high-sensitivity, minimally invasive assays, such as skin-swab based qPCR/ddPCR or validated host biomarker tests-at peripheral health centers would support early detection, treatment monitoring, and robust surveillance, ultimately reducing transmission from dermal reservoirs. Implementation research should improve case detection, adherence, stigma reduction, and integration of PKDL surveillance into VL elimination programs.
